# Successfully performed video capsule endoscopy in an 8‐month‐old infant weighing 7.5 kg

**DOI:** 10.1002/jpr3.70181

**Published:** 2026-04-06

**Authors:** Paul‐Christoph Zeisler, Robert Thimme, Ulrike Teufel‐Schaefer, Michael Schultheiß

**Affiliations:** ^1^ Department of Medicine II Medical Center – University of Freiburg Freiburg Baden‐Württemberg Germany; ^2^ Department of General Pediatrics Adolescent Medicine and Neonatology, Medical Center – University of Freiburg Freiburg Baden‐Württemberg Germany

**Keywords:** children, PillCam, small bowel bleeding, small bowel capsule endoscopy, XIAP‐deficiency

## Abstract

Video capsule endoscopy (VCE) is a well‐established diagnostic tool for examining the small bowel. Limited data exist on its use in infants. To our knowledge, we present the first detailed case of a successful PillCam®SB3‐VCE performed in an 8‐month‐old infant weighing 7.5 kg with suspected small bowel bleeding following allogeneic hematopoietic stem cell transplantation. The capsule was endoscopically placed into the duodenum without prior bowel cleansing or patency capsule testing. VCE identified bleeding in the terminal ileum and cecum. This case demonstrates that VCE can be safely performed even in very young, low‐weight infants when clinically necessary. However, due to anatomical and physiological challenges, VCE should be carefully considered in infants and applied cautiously on a case‐by‐case basis.

## 1 INTRODUCTION

Video capsule endoscopy (VCE) is a recognised tool for small bowel examination. VCE is approved by the U.S. Food and Drug Administration (FDA) for use in children over 2 years of age. However, limited data are available regarding its use in younger children.

The main concern when using VCE in children is the risk of capsule retention. To date, the youngest reported patient to successfully undergo VCE was 8‐months‐old.^1^

To our knowledge, we present the first detailed case report of a safely performed PillCam®SB3‐VCE in an 8‐month‐old patient with suspected small bowel bleeding. This is not only the youngest but also the lowest‐weight patient to receive a VCE to date.

## 2 CASE REPORT

VCE was performed in an 8‐month‐old patient with suspected small bowel bleeding following allogeneic hematopoietic stem cell transplantation (HSCT).

A few months after birth, the patient was diagnosed with XIAP‐deficiency (ORPHA:538934). Initially, he was treated with dexamethasone; however, 1 month later, he developed a relapse of hemophagocytic lymphohistiocytosis (HLH). So therapy was escalated using dexamethasone, etoposide, tocilizumab and ruxolitinib. Due to the severe disease progression, HSCT was recommended. Since no HLA‐matched unrelated donor was found, umbilical cord blood from a donor has been used.

Conditioning therapy consisted of fludarabine, melphalan, thiotepa, alemtuzumab and hydroxyurea with doses adjusted to body weight. Graft‐versus‐host disease prophylaxis included tacrolimus and mycophenolate mofetil (MMF). In addition, anti‐infective and veno‐occlusive disease prophylaxis were administered. Neutrophile engraftment occurred on Day 21 posttransplant.

On Day 27 posttransplant, the patient developed severe gastrointestinal bleeding with melena, requiring repeated blood transfusions. Treatment with tranexamic acid was ineffective.

On Day 31 posttransplant, MMF was discontinued, as gastrointestinal bleeding is a known adverse effect of the drug.

On Day 32 posttransplant (Table 1), a gastroscopy and colonoscopy were performed; however, no active bleeding was detected. No biopsies were taken. Immediately afterwards, a VCE was conducted, allowing bedside examination of the small bowel, without the need for transportation to the radiology department and allowing real‐time visualisation through the recorder's integrated monitor. No specific bowel cleansing preparation was performed, as the patient received only parenteral nutrition. A patency capsule was not used before the procedure. However, ultrasound and clinical examination were conducted to rule out obstruction. The PillCam®SB3 capsule (Medtronic) and the standard adult sensor belt were used.

**Table 1:** Haemoglobin levels and platelet counts the days around VCE.

Time post HSCT (days)	Time (hh:mm)	Haemoglobin (g/dL)	Platelet counts (g/L)
+27	07:45 AM	8.8	88
+27	02:30 PM	7.9	56
+28	07:30 AM	13.8	38
+28	01:45 PM	12.7	39
+29	06:00 AM	8.9	118
+31	07:45 AM	10.6	62
+31	01:45 PM	6.8	73
+32	07:00 AM	10.4	140
+32	10:30 AM	7.7	98
+32	02:30 PM	10.0	57
+33	06:30 AM	7.4	190
+34	06:15 AM	11.3	162
+35	07:45 AM	11.7	187
+38	06:00 AM	10.6	79

*Note*: (green: day VCE performed).

Abbreviations: HSCT, hematopoietic stem cell transplantation; VCE, video capsule endoscopy.

**Figure 1:**
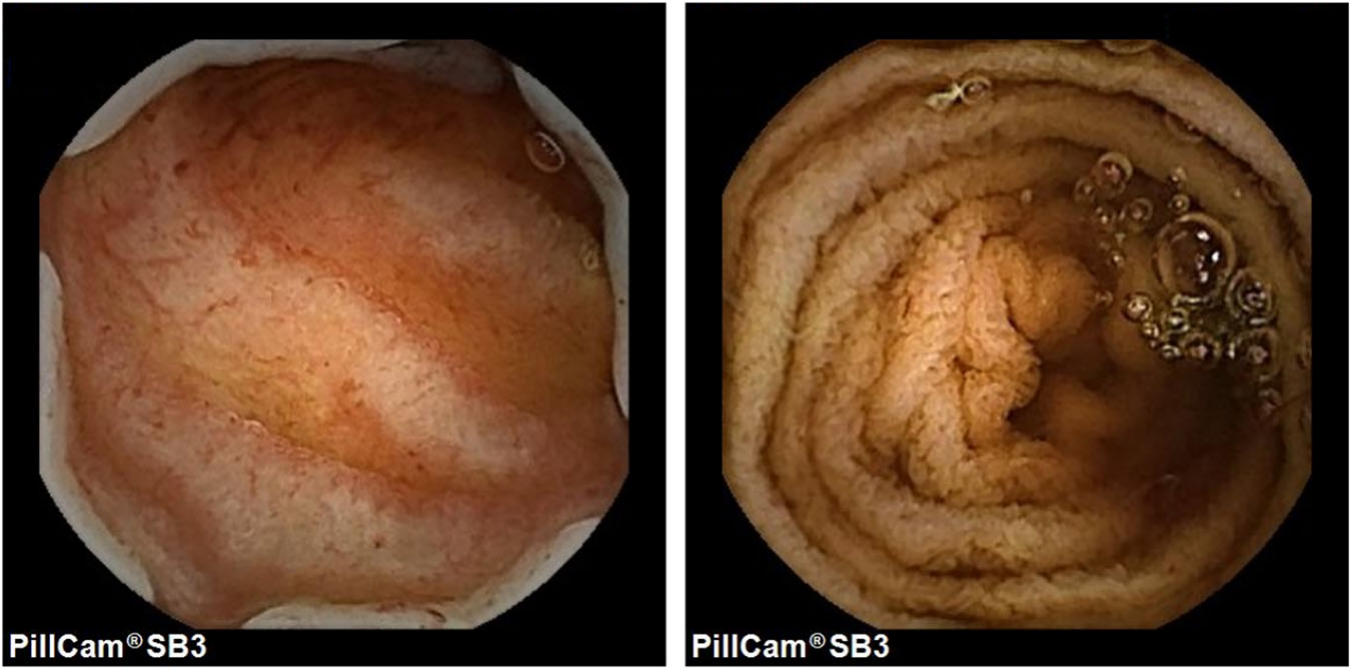
Left: capsule safely secured in the AdvanCE delivery device introducer moments before release in the duodenum. Right: normal appearance of the small bowel mucosa.

At the time of VCE, the patient measured 58 cm in height and weighed 7485 g. The capsule was placed into the duodenum using the AdvanCE delivery device introducer (STERIS) (Figure 1). Duodenal release occurred 07:49 min after capsule activation. The small bowel transit time (SBTT) was 423 min.

**Figure 2:**
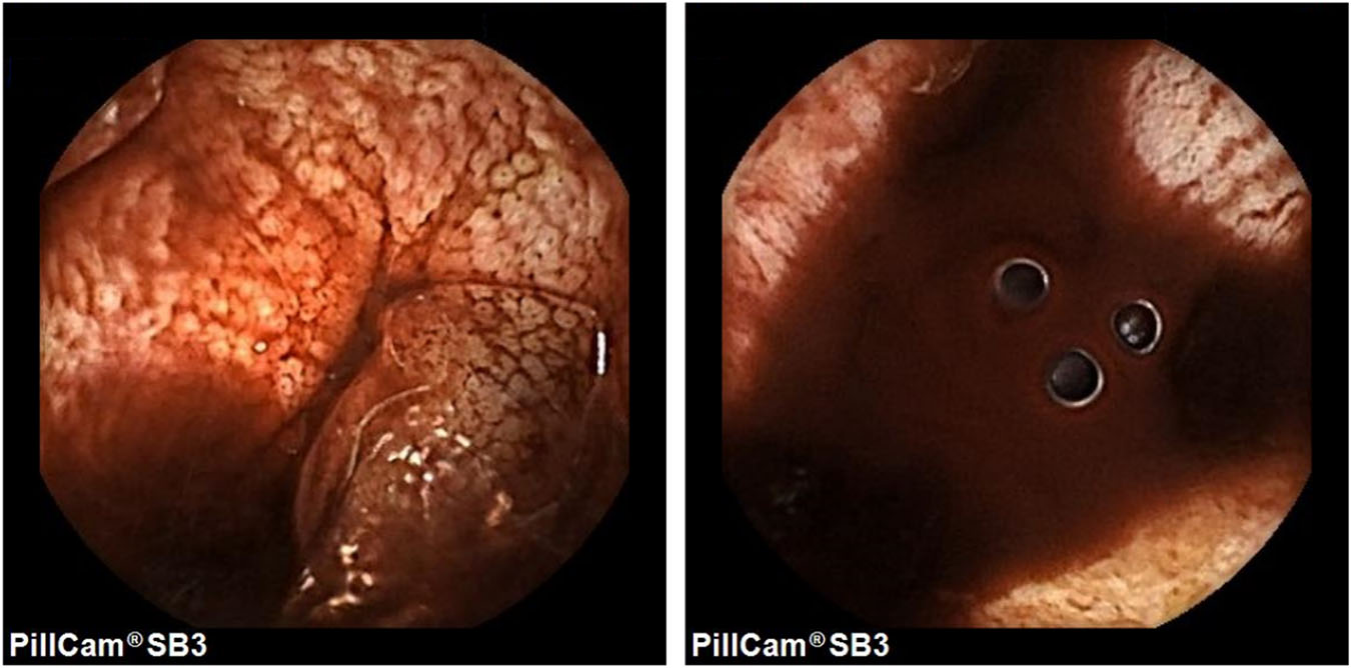
Active bleeding detected in the ileocecal region (left, right).

VCE revealed bleeding in the terminal ileum and cecum (Figure 2). No other abnormalities were detected. With MMF paused, the bleeding resolved and haemoglobin levels stabilised. However, platelet count remained low, requiring repeated transfusions. Eltrombopag was administered off‐label due to severe gastrointestinal bleeding in the past and persistent thrombocytopenia.

In total, 26 red blood cell concentrates and 77 platelet concentrates were administered. The patient was discharged 88 days after HSCT.

## 3 DISCUSSION

XIAP‐deficiency is a rare inherited immunodeficiency characterised by a dysregulated immune response, often triggered by Epstein‐Barr virus infection. The prevalence is less than 1 in 1,000,000. Clinical presentation is diverse and may include HLH, hepatitis, splenomegaly, hypogammaglobulinemia and colitis.

VCE enables noninvasive visualisation of the small bowel and is FDA‐approved for children over 2 years. Only a few cases of VCE have been reported in younger patients. In a large paediatric cohort undergoing VCE, the youngest patient was 8‐month‐old.^1^ We present the first detailed case report of a successful PillCam®SB3‐VCE in an infant of this age.

The main indication for VCE in children is Crohn's disease, in children below the age of 8 years, suspected bleeding is the most frequent indication for VCE.^2^, ^3^ In our case, the indication was a suspected small bowel bleeding following HSCT.

Our patient measured 58 cm in height and weighed 7485 g. PillCam®SB2‐VCE was safely performed in a child weighing 7.9 kg.^4^ The other 8‐month‐old child weighed 9 kg.^1^ Therefore, our case presents a successfully performed PillCam®SB3‐VCE in the patient with the lowest weight reported to date.

Primary concerns with paediatric VCE are capsule retention and aspiration; the minimum safe age and weight have yet to be established in randomised trials.

There is no clear evidence that children have a higher risk of capsule retention compared to adults. A meta‐analysis reported a retention rate of 2.3% in children.^3^ However, in a multicentre study, no capsule retention was observed in children under 8 years of age.^5^ If retention occurs, endoscopic or surgical retrieval appears to carry a higher risk of complications in children than in adults; therefore, extra caution is required.

The capsule diameter was 11.4 mm. The diameter of the small intestine varies and increases with age (mean diameter 13.0 mm at the age of 1 year).^6^ It has been demonstrated that dummy capsules can pass through the ileocecal valve in children as young as 6 months.^5^ Based on these dimensions, the capsule should pass through the small bowel without difficulties. However, these data represent averages and may not account for individual variation, especially in infants after HSCT. In our case, the small bowel diameter did not appear to affect the quality of the examination.

The capsule was placed endoscopically. Capsule activation to duodenal delivery time aligned with literature.^1^ The capsule could be placed into the duodenum without difficulty, causing only minor mucosal lesions. In contrast, a 14‐month‐old patient required endoscopic dilatation of the pylorus before the capsule could be placed into the duodenum.^1^

Voluntary capsule‐swallowing remains challenging, particularly in younger children. Successful capsule‐swallowing has been reported even in children as young as 4 years old.^5^ If a child is unable to swallow the capsule, endoscopic placement using specialised devices—such as those use in our case—is necessary.

The SBTT was 423 min. This aligns with previously published SBTT in young children.^4^ However, another study found no significant age‐related difference in children in SBTT.^1^ The SBTT in our case was longer than the mean times described in literature for both children and adults.^1^, ^7^, ^8^

No small bowel cleansing solution was administered; nevertheless, an acceptable level of mucosal cleanliness was attained.

## 4 CONCLUSION

Our case demonstrates VCE can be safely performed in an 8‐month‐old infant under 7.5 kg. However, due to size and anatomy, the risk of capsule retention may be higher. Thus, VCE should remain an ‘ultima ratio’ diagnostic tool in young children, reserved for critical cases.

## CONFLICT OF INTEREST STATEMENT

Paul‐Christoph Zeisler: Travel sponsoring Medtronic GmbH. Robert Thimme: none. Ulrike Teufel‐Schaefer: none. Michael Schultheiß: Lecture fees: Falk Foundation e.V., W. L. Gore & Associates. Consultant fees: Bentley InnoMed, Bristol‐Myers Squibb GmbH.

## ETHICS STATEMENT

Informed patient consent was obtained from parents for publication of the case.
